# Landscape Analysis of Matrix Metalloproteinases Unveils Key Prognostic Markers for Patients With Breast Cancer

**DOI:** 10.3389/fgene.2021.809600

**Published:** 2022-01-06

**Authors:** Tianyi Cheng, Peiying Chen, Jingyi Chen, Yingtong Deng, Chen Huang

**Affiliations:** ^1^ Faculty of Chinese Medicine, State Key Laboratory of Quality Research in Chinese Medicines, Macau University of Science and Technology, Macau, China; ^2^ Dr. Neher’s Biophysics Laboratory for Innovative Drug Discovery, Macau University of Science and Technology, Macau, China

**Keywords:** breast cancer, matrix metalloproteinases, immune infiltration, bioinformatics, prognostic value

## Abstract

Breast cancer (BRCA) is the most common cancer in the world, of which incidence rate and mortality are the highest in women. Being responsible for the remodeling and degradation of extracellular matrix proteins, matrix metalloproteinases (MMPs) have been regarded as one of the most important protease family related to tumorigenesis. It has been demonstrated that MMPs play crucial roles in some tumor invasion and metastasis. However, the potential roles of MMPs in tumorigenesis and progression of BRCA and its subtype remain elusive. Herein, we conducted a systematic study on MMPs *via* a series of database-based retrospective analysis, including TCGA, R Studio, GEPIA, Kaplan-Meier Plotter, cBioPortal, STRING, GeneMANIA and TIMER. As a result, many MMP family members were differentially expressed in patients with BRCA, e.g., the expressions of MMP1, MMP9, MMP11 and MMP13 were up-regulated, whereas the expression levels of MMP19 and MMP28 were down-regulated. MMP9, MMP12, MMP15 and MMP27 were significantly correlated with the clinical stages of BRCA, implying their important roles in the occurrence and development of BRCA. In addition, the survival analysis indicated that different expression pattern of MMPs exhibited distinct outcomes in patient with BRCA, e.g., patients with high expression of MMP2, MMP8, MMP16, MMP17, MMP19, MMP20, MMP21, MMP24, MMP25, MMP26 and MMP27 had a prolonged survival time, while the others (MMP1, MMP7, MMP9, MMP12 and MMP15) exhibited poor prognosis. Subsequent functional and network analysis revealed MMPs were mainly correlated with parathyroid hormone synthesis and secretion pathway, collagen metabolism, and their effect on the activities of serine hydrolase, serine peptidase and aminopeptidase. Notably, our analysis showed that the expression of MMPs was significantly correlated with the infiltration of various immune cells in BRCA, including CD8+T cells, CD4+T cells, macrophages, neutrophils, B cells, and dendritic cells, suggesting the close correlations between MMPs and immune functions. In short, our study disclosed MMPs play multiple biological roles in the development of BRCA, MMP1 and MMP9 might be used as independent prognostic markers and potential therapeutic targets for diagnosis and treatment for patients with BRCA.

## 1 Introduction

### 1.1 Background

In 2020, breast cancer (BRCA) has become the most common cancer worldwide, replacing lung cancer (Sung et al., 2021). Five major intrinsic subtypes of breast tumors were identified, including Basal-like, Luminal A, Luminal B, Human epidermal growth factor receptor 2-positive/estrogen receptor-negative (HER2+/ER−) and Normal Breast-like (Zhang et al., 2018). Although BRCA diagnostic examinations such as mammography, computed tomography, magnetic resonance imaging, biopsy, ultrasound, and molecular imaging had made a significant progression, mortality is still progressively increasing because BRCA is undetected in initial stages and breast tumor can metastasize (Perez-Rivas et al., 2012). Early BRCA has no obvious characteristics, which is often ignored by patients and makes them blunder away the most appropriate treatment time (Ren et al., 2020). The limitation relates to the evidence that available tumor markers show low levels of sensitivity (Lawicki et al., 2016), which makes the necessity of potential constructive diagnostic methods of detection. Additionally, previous studies have shown that the major reason for the death of BRCA patients is the invasion and metastasis of breast tumors (Curigliano et al., 2017). Therefore, mechanisms and drugs that effectively inhibit tumor cell migration and invasion have become hotspots (Luo and Zhou 2020).

Matrix metalloproteinases (MMPs) are a family of zinc-dependent endoproteases responsible for the tissue remodeling and degradation of extracellular matrix (ECM) proteins (Nagase et al., 2006). MMPs are the most important protease family related to tumorigenesis (Kessenbrock et al., 2010), and play an important role in tumor invasion and metastasis (Endres et al., 2016). There are at least 23 matrix metalloproteinases expressed in human body, and they can usually be divided into the following five types (Cerofolini et al., 2019): 1) Non-furin regulated MMPs, including MMP1, MMP3, MMP7, MMP8, MMP10, MMP12, MMP13, MMP20 and MMP27; 2) MMPs containing three fibronectin-like inserts in the catalytic domain, including MMP2 and MMP9; 3) MMPs anchored to the cell membrane via the C-terminal glycosylphosphatidylinositol moiety, including MMP11, MMP17 and MMP25; 4) MMPs with transmembrane domains, including MMP14,MMP15, MMP16 and MMP24; 5) Other types of MMPs, including MMP19, MMP21, MMP23, MMP26 and MMP28.

In previous studies, some genes of the MMP family have been confirmed to be related to breast cancer. For example, MMP2 and MMP9 were possible tumor markers for breast cancer patients (Stankovic et al., 2010; Radenkovic et al., 2014). MMP7 was a decisive factor in Chinese women with breast cancer (Beeghly-Fadiel et al., 2009). The level of MMP7 in patients with bone metastasis was much higher than that in patients without bone metastasis (Voorzanger-Rousselot et al., 2006). The expression level of MMP11 in BRCA was higher than that in normal tissues, reflecting the differentiation stage of BRCA, and could be used as one of the prognostic markers of breast cancer (Cheng et al., 2010). Some scholars also investigated that MMP14 was the most significant factor related to the prognosis of breast cancer (Têtu et al., 2006). However, the functions and prognostic effects of other different types of MMP family members in breast cancer tissues are still unknown. Therefore, this study aims at unveiling clinical values of MMPs in BRCA and its subtypes via analyzing the expression differences, prognostic effects, gene mutations, as well as their potential association with immune cell infiltration level based on the application of series of public biological data platforms.

## 2 Methods

### 2.1 The Gene Expression Profiling Interactive Analysis Database Used to Extract the Expression of the Matrix Metallo Proteinases Family

The GEPIA database (Gene Expression Profiling Interactive Analysis, http://gepia.cancer-pku.cn/) contains RNA sequencing data of common malignant tumor samples and normal samples in the TCGA and GEO databases (Tang et al., 2017). In this study, the GEPIA database was used to extract the expression and pathological stage analysis of each gene of the MMP family in breast cancer. For the expression analysis, we selected the “expression analysis” mode and “BRCA” as cancer types. Each MMP was input. Other options were set to the default values. For the pathological stage analysis, we input each MMP gene, added “BRCA” as cancer types, and used major stage for plotting.

### 2.2 Kaplan-Meier Plotter Database Used to Analyze the Relationship Between Matrix Metallo Proteinases and Breast Cancer Survival and Prognosis

This study used the Kaplan-Meier Plotter data analysis platform (Nagy et al., 2018) (https://kmplot.com/analysis/) to search for each gene in the MMP family, for survival analysis of breast cancer patients. In this analysis, the pathological type, clinical staging, and grading conditions of breast cancer were not restricted.

### 2.3 cBioPortal Used to Analyze the Expression of Matrix Metallo Proteinases in Breast Cancer

cBioPortal (cBio Cancer Genomics Portal, http://cbioportal.org/) provides a multi-dimensional visualization tool for research and analysis of cancer-related genetic data (Gao et al., 2013). Based on 283 large-scale tumor-related gene expression and proteomics studies in databases such as TCGA, gene mutation analysis of MMPs in the cBioPortal database was conducted, along with the survival rate analysis of the mutant group and the non-mutant group.

### 2.4 GeneMANIA Database Used for Function Analysis of Matrix Metallo Proteinases

The GeneMANIA database (Warde-Farley et al., 2010) (http://genemania.org/) generates hypotheses about gene functions and analyzes gene lists, and determines the priority of genes according to their functions. The list of MMP family genes was imported. Functional analysis of differentially expressed MMP family genes and related molecules was performed.

### 2.5 STRING Database Used to Analyze Protein-Protein Interactions of Matrix Metallo Proteinases

The STRING database (Szklarczyk et al., 2019) (https://www.string-db.org/) is a platform for analyzing protein interactions. The database was used to analyze the interaction between known proteins and predicted proteins, including direct interactions and indirect interactions. This study analyzed the interaction of MMP family proteins through STRING database. The protein-protein interactions (PPI) network was constructed from the STRING database (https://string-db.org/), which included data compiled from several sources. “MMP1, MMP2, MMP3, MMP7, MMP8, MMP9, MMP10, MMP11, MMP12, MMP13, MMP14, MMP15, MMP16, MMP17, MMP19, MMP20, MMP21, MMP24, MMP25, MMP26, MMP27 and MMP28” were input to the “multiple proteins” box with “*Homo sapiens*” selected as the organism. Other options were left as default options.

### 2.6 TIMER Database Used for Analysis of the Immune Infiltration Status of Matrix Metallo Proteinases

The TIMER2.0 (Li et al., 2017) (http://timer.comp-genomics.org/) database was used to systematically evaluate the infiltration of different immune cells and their clinical effects. This study analyzed the correlation between MMP expression in BRCA and the infiltration of six immune cells (CD4^+^T cells, CD8^+^T cells, B cells, macrophages, neutrophils, and dendritic cells in breast cancer) through the TIMER2.0 database.

### 2.7 The Cancer Genome Atlas and R Studio Used for Analysis of the Breast Cancer Subtype

The Cancer Genome Atlas (TCGA) (https://portal.gdc.cancer.gov/) is a genomics database and used for analyzing cancer samples (Tomczak et al., 2015). To comprehensively investigate corresponding analysis of MMPs and BRCA subtypes, we used R package Kruskal wallis and ggplot2 to analyze the differential expression of MMPs in five major subtypes of BRCA. We also used R package cgdsr, survival and survminer to analyze the survival and prognosis for each subtype. Moreover, we investigated the estimate score of MMP1 and MMP9 in five subtypes of BRCA via R Package Estimate.

### 2.8 Statistical Methods

The Kaplan-Meier model was used to analyze the relationship between breast cancer survival prognosis and the expression of MMP family, and the log-rank test was used to estimate the difference in survival rate. The MMP family genes were significantly correlated with immune cell infiltration (*p* < 0.05) through the GENE module in the TIMER2.0 database.

## 3 Results

### 3.1 Differential Expression of Matrix Metallo Proteinases in Breast Cancer and its Subtype Patients

**FIGURE 1:**
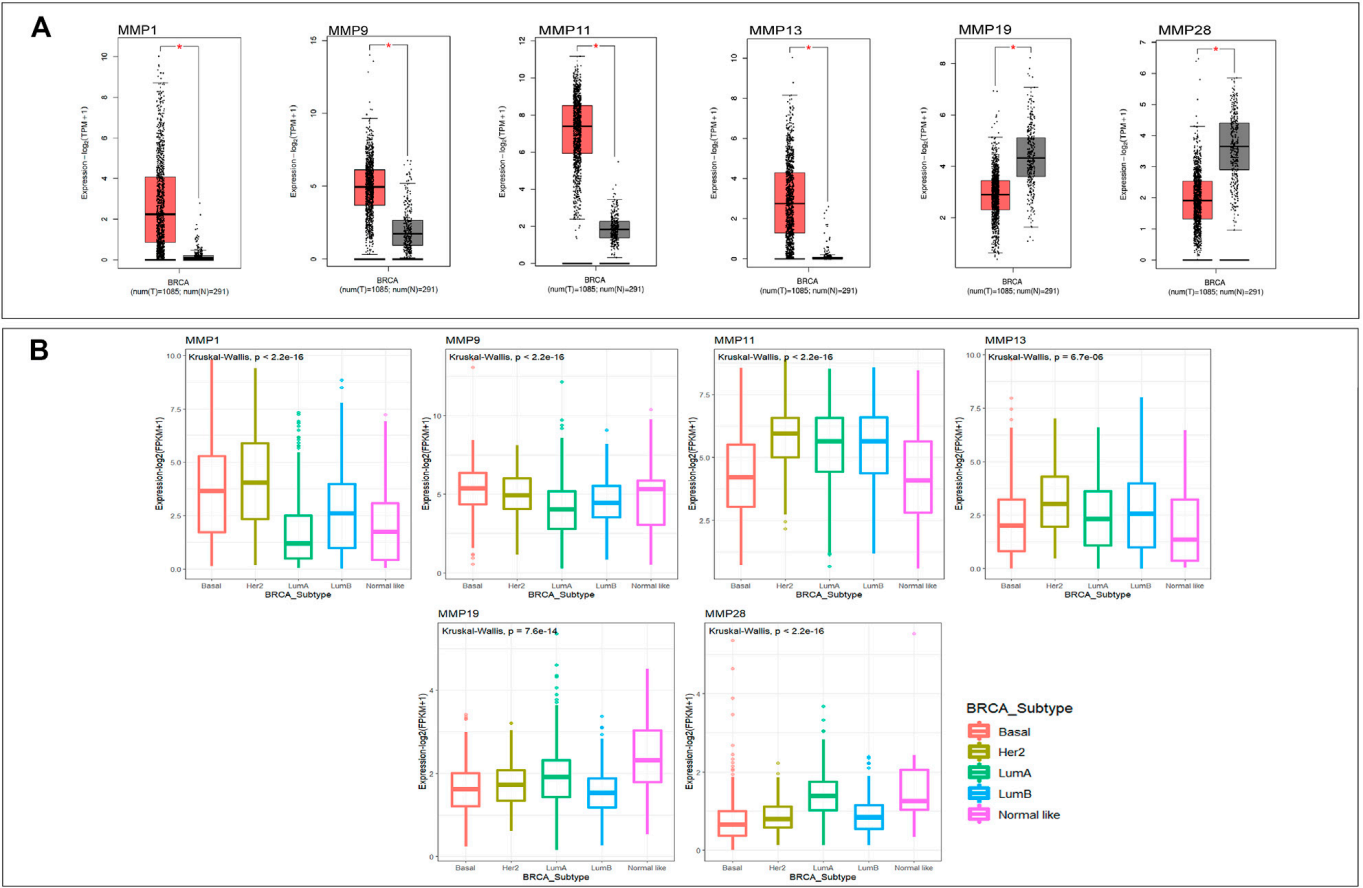
**(A)**The mRNA expression of MMPs in breast cancer and normal breast tissue (GEPIA). The expression levels of MMP1, MMP9, MMP11, and MMP13 were higher in breast cancer tissues than in normal tissues, and the expression levels of MMP19 and MMP28 were lower in the former than the latter (*p* value <0.05). **(B)** The mRNA expression of MMPs in five subtypes of breast cancer (*p* value <0.05). The expression levels of MMP1, MMP9, MMP11, and MMP13 in Her2 subtype were the highest. The expression levels of MMP19 and MMP28 in Basal and LumB subtype were lower than that in other.

Through the GEPIA database, we compared the mRNA expression of MMPs in tumor with normal breast tissues, including 1085 BRCA samples and 291 normal tissue samples. The results showed that the expression levels of MMP1, MMP9, MMP11, and MMP13 were significantly up-regulated (*p* < 0.05) in BRCA (Figure 1A) The expression levels of MMP19 and MMP28, however, in BRCA were significantly decreased compared with normal samples (*p* < 0.05) (Figure 1A). The expressions of other genes in the MMP family in BRCA exhibited no statistically difference. Additionally, we further explored the differential expression of MMPs in the five subtypes of BRCA by using R package Kruskal wallis and ggplot2 (Figure 1B). In Her2 subtype, the expression levels of MMP1, MMP9, MMP11, and MMP13 were highest. In Basal and LumB subtype, the expression levels of MMP19 and MMP28 were lower than others (*p* < 0.05).

### 3.2 Correlation Analysis Between Matrix Metallo Proteinases and Pathological Stages of Breast Cancer

**FIGURE 2:**
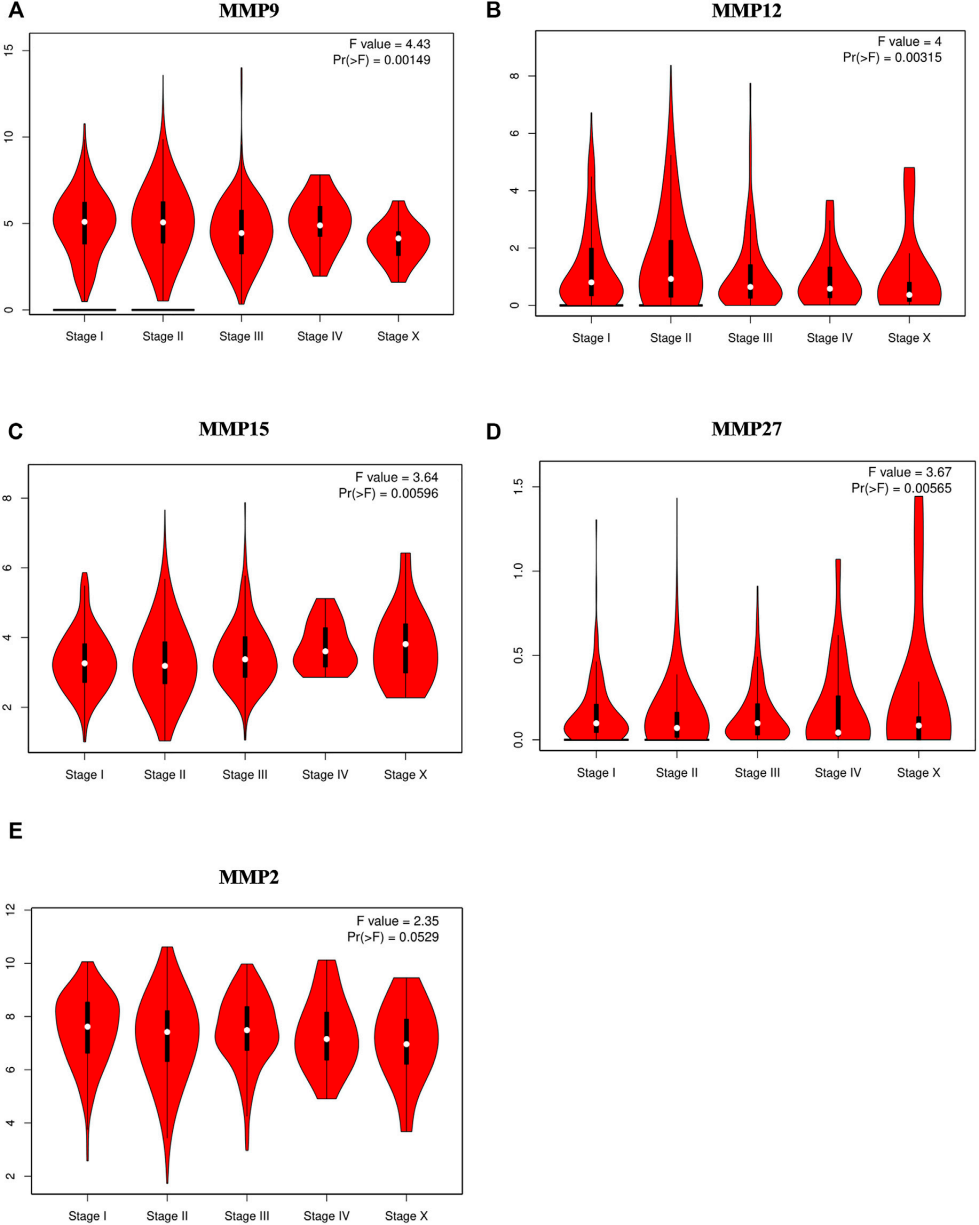
Correlations between MMPs expression and tumor stage in breast cancer patients (GEPIA). **(A,B,C,D)** The expression of MMP9, MMP12, MMP15, MMP27 was correlated with the pathological stage of breast cancer patients (*p* < 0.05). **(E)** The *p* value of MMP2 was close to 0.05 (*p* = 0.0529) whose significance needs further study.

Next, we want to investigate the potential correlation between the expression of MMPs and the pathological stages in BRCA. Similarly, GEPIA was used to achieve this analysis. The results showed that MMP9, MMP12, MMP15, and MMP27 groups were all highly variable (*p* < 0.01), whereas the other groups were not markedly different, indicating that these genes may play important roles in the occurrence and development of breast cancer (Figures 2A–D). Notably, the difference of MMP2 among five stages was close to 0.05 (*p* = 0.0529), which suggested that its significance may need further studies (Figure 2E).

### 3.3 Prognosis of the Expression of Matrix Metallo Proteinases in Breast Cancer and its Subtype Patients

**FIGURE 3:**
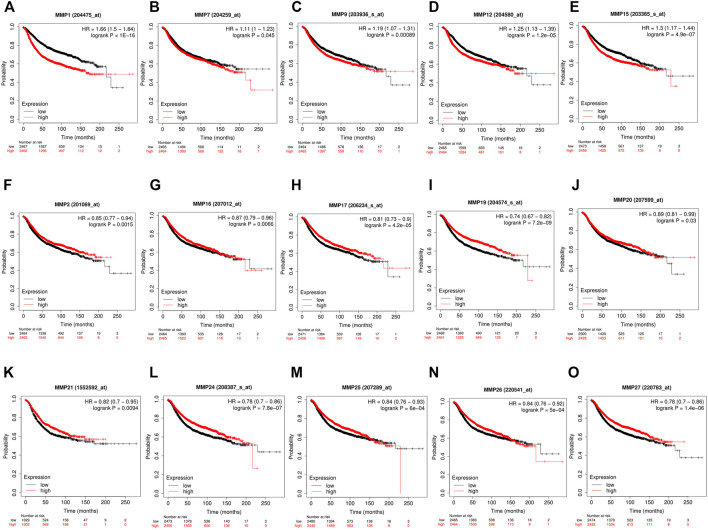
The recurrence-free survival (RFS) of MMPs (Kaplan-Meier Plotter). **(A,B,C,D,E)** High mRNA expression of MMP1, MMP7, MMP9, MMP12 and MMP15 was correlated with a poorer prognosis, and **(F,G,H,I,J,K,L,M,N,O)** low expression of MMP2, MMP8, MMP16, MMP17, MMP19, MMP20, MMP21, MMP24, MMP25, MMP26 and MMP27 was correlated with a poorer prognosis in breast cancer patients (*p* < 0.05).

Our above-mentioned analyses indicated that several MMP genes were significantly expressed in BRCA. In order to further clarify the prognostic values of these MMPs in patients with BRCA, the Kaplan-Meier Plotter database was used to analyze the recurrence-free survival (RFS) and overall survival (OS) of the genes of the MMP family. The results showed that the expression of many MMPs exhibited correlation with the RFS of BRCA patients. Concretely, patients with high MMP1, MMP7, MMP9, MMP12, and MMP15 were correlated with short RFS compared to low MMP mRNA expression (*p* < 0.05) (Figures 3A–E), whereas the patients with lower expression of MMP2, MMP8, MMP16, MMP17, MMP19, MMP20, MMP21, MMP24, MMP25, MMP26, and MMP27 had a poor survival time (*p* < 0.05) (Figure 3F–O).

**FIGURE 4:**
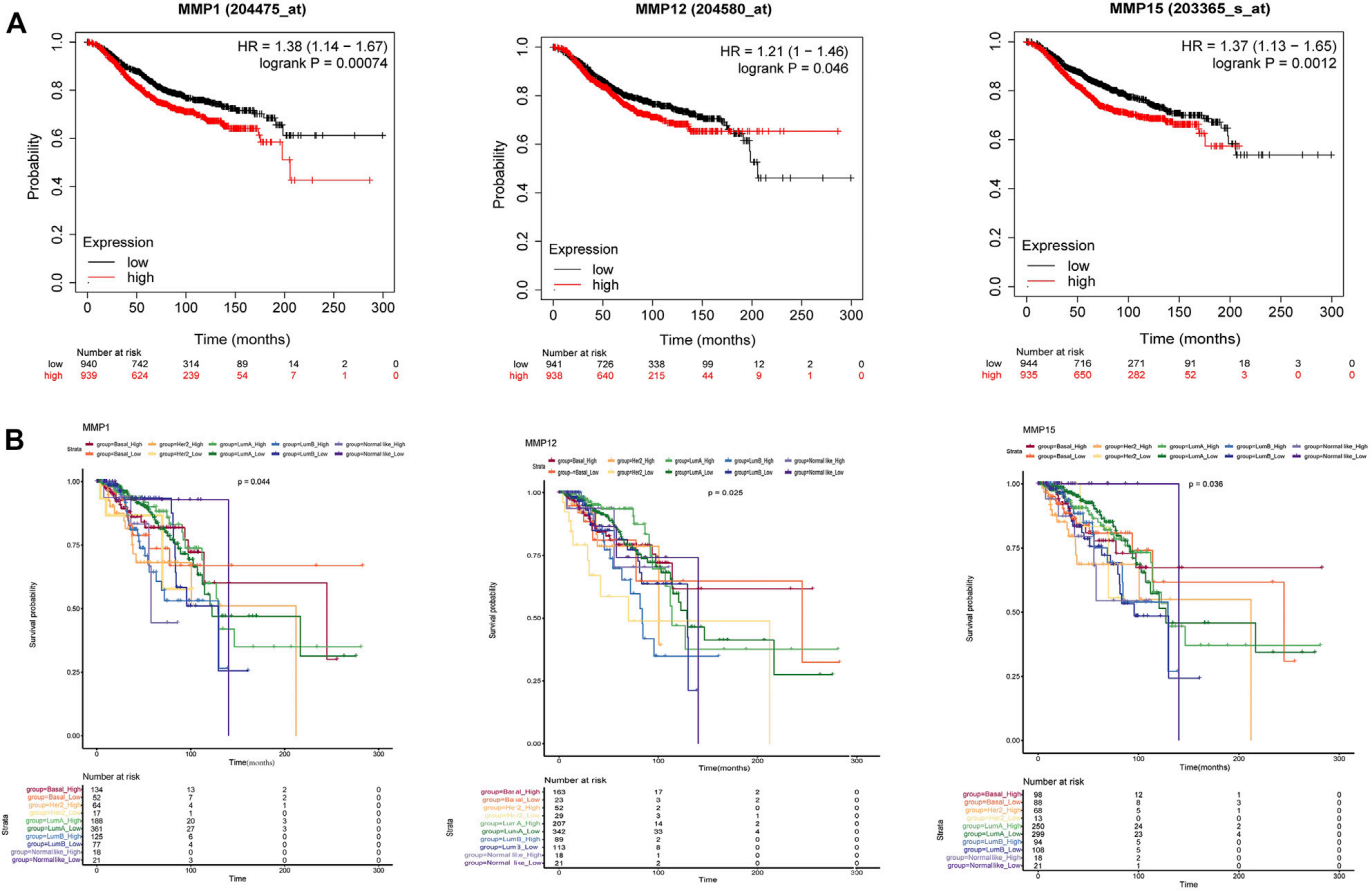
**(A)** The overall survival of BRCA in MMP1, MMP12 and MMP15. High expression of MMP1, MMP12 and MMP15 was correlated with a poorer prognosis (*p* < 0.05). (Kaplan-Meier Plotter) **(B)** The overall survival of five BRCA subtypes in MMP1, MMP12 and MMP15 (*p* < 0.05).

As for the OS analysis of BRCA and its subtype, it was indicated that the overall survival rate of BRCA patients with high expression of MMP1, MMP12, MMP15 were significantly lower than that of patients with low expression (Figure 4A) (*p* < 0.05). The remaining OS analysis showed no significant statistical difference (*p* > 0.05). Moreover, we explored the survival and prognosis for each subtype (Figure 4B). There were statistical significances of overall survival between five subtypes and the high expression of MMP1, MMP12 and MMP15. Notably, the high expression of MMP15 had a poorer prognosis in Normal-like subtype than that in other subtypes (*p* = 0.033).

### 3.4 Matrix Metallo Proteinases Related Gene Mutation, Expression and Interaction Analysis

**FIGURE 5:**
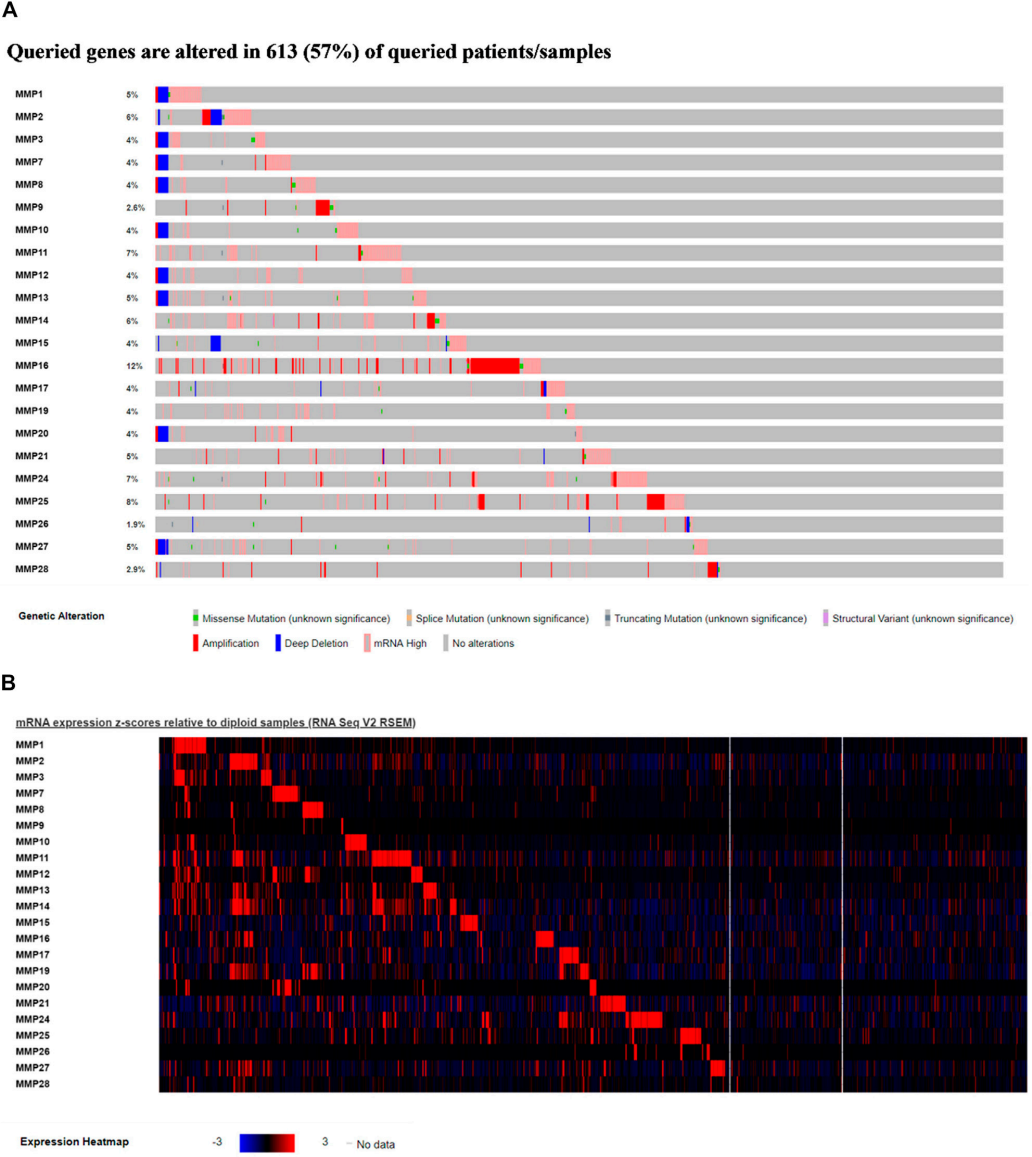
**(A)**Waterfall map of alterations in different expressed MMPs in BRCA. **(B)** Heat map of alterations in different expressed MMPs in BRCA.

To further explore, we extracted genomic alterations by Expectation-Maximization (RSEM) value for every MMP gene in the BRCA-TCGA cohort using RNA seq values obtained from cBioPortal. In this study, a waterfall map and heat map of tumor gene mutations of the remaining genes were conducted (Figure 5), and it was found that 57% of the 1,084 samples, namely 613 cases, had genetic mutations. Among them, MMP17, MMP24, MMP25, MMP11, MMP2, MMP14 were the most common genes that had changed, and their mutation rates were 12, 8, 7, 7, 6, and 6%, respectively.

**FIGURE 6:**
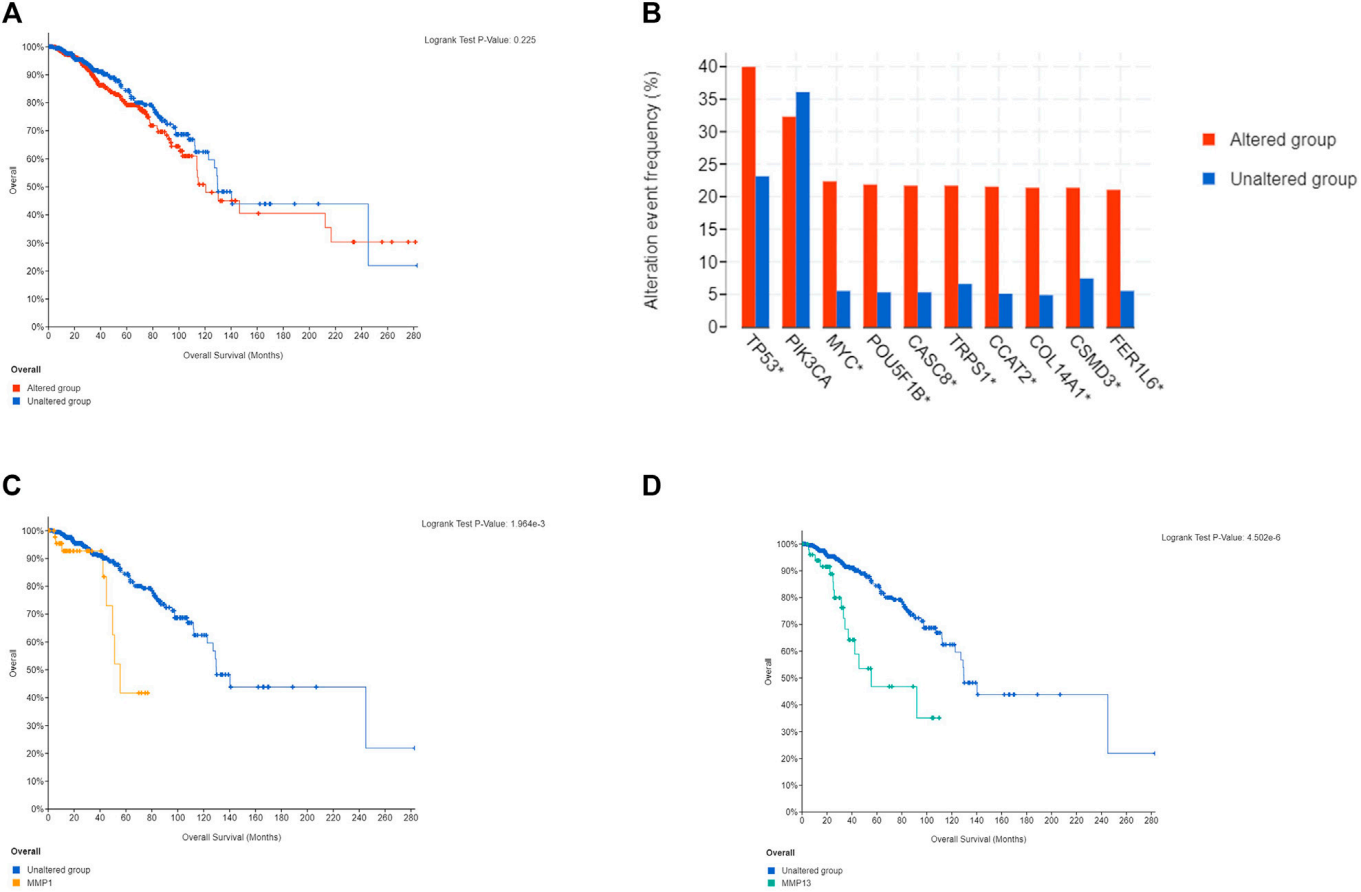
Histogram of genes with the highest frequency in the genome and MMPs survival curve. **(A)** Overall survival curve of MMP family. **(B)** Histogram of genes with the highest frequency in the genome. **(C)** MMP13 overall survival curve. **(D)** MMP1 overall survival curve.

In the meanwhile, we found that in the matrix metalloproteinase mutation group, tumor protein (TP53) and phosphatidylinositol-3-kinase catalytic subunit *α* (PIK3CA) had the most abundant gene mutations (Figure 6B). Secondly, we conducted survival analysis between the mutant group and the wildtype group. The results showed that there was no significant statistical difference between the mutant group and the wildtype group (Figure 6A), while the overall survival rate in mutant MMP1 and MMP13 group was lower than the unaltered group (Figures 6C,D), which suggested that the patient with mutant MMP1 and MMP13 had a poor prognosis.

**FIGURE 7:**
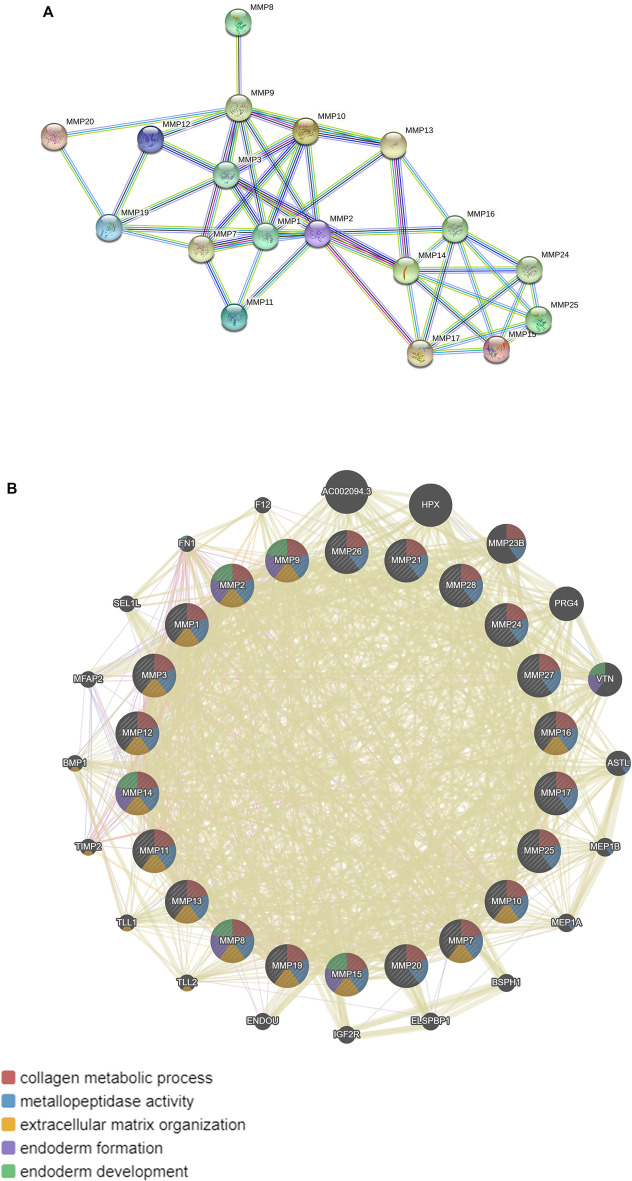
Protein-protein mutual aid and interaction analysis diagram of MMPs. **(A)** Protein protein mutual aid analysis diagram of MMPs (String). **(B)** Protein protein interaction analysis diagram of MMPs and its related molecules (GeneMANIA).

In addition, MMPs were subjected to STRING for PPI analysis, the species was set to “*Homo sapiens*”, and the other values were kept as default settings for PPI analysis. Our results showed that the PPI network had 22 nodes and 50 edges, and the average node degree was 4.55. The average local clustering coefficient was 0.519. The expected number of edges was 2. The PPI enrichment *p*-value was less than 1.0e-16 (Figure 7A). Furthermore, KEGG pathway enrichment analysis obtained from STRING database showed enrichment function in parathyroid hormone synthesis, secretion and action, IL-17 signaling pathway, relaxin signaling pathway (Table 1).

**TABLE 1:** KEGG pathway enrichment of MMPs (STRING).

Description	Count in Network	Strength	False discovery rate
Parathyroid hormone synthesis, secretion and action	7 of 103	1.62	1.97e-07
IL-17 signaling pathway	5 of 92	1.52	0.00011
Relaxin signaling pathway	5 of 128	1.38	0.00026

Moreover, we used GeneMANIA database to analyze MMPs again and the results showed that MMPs and related molecules, such as: AC002094.3, HPX, PRG4, VTN, and the function of differentially expressed genes were mainly related to collagen metabolism process, serine hydrolase, serine peptidase, and the activity of aminopeptidase (Figure 7B).

### 3.5 Immune Cell Infiltration of Matrix Metallo Proteinases in Breast Cancer and its Subtype Patients

**FIGURE 8:**
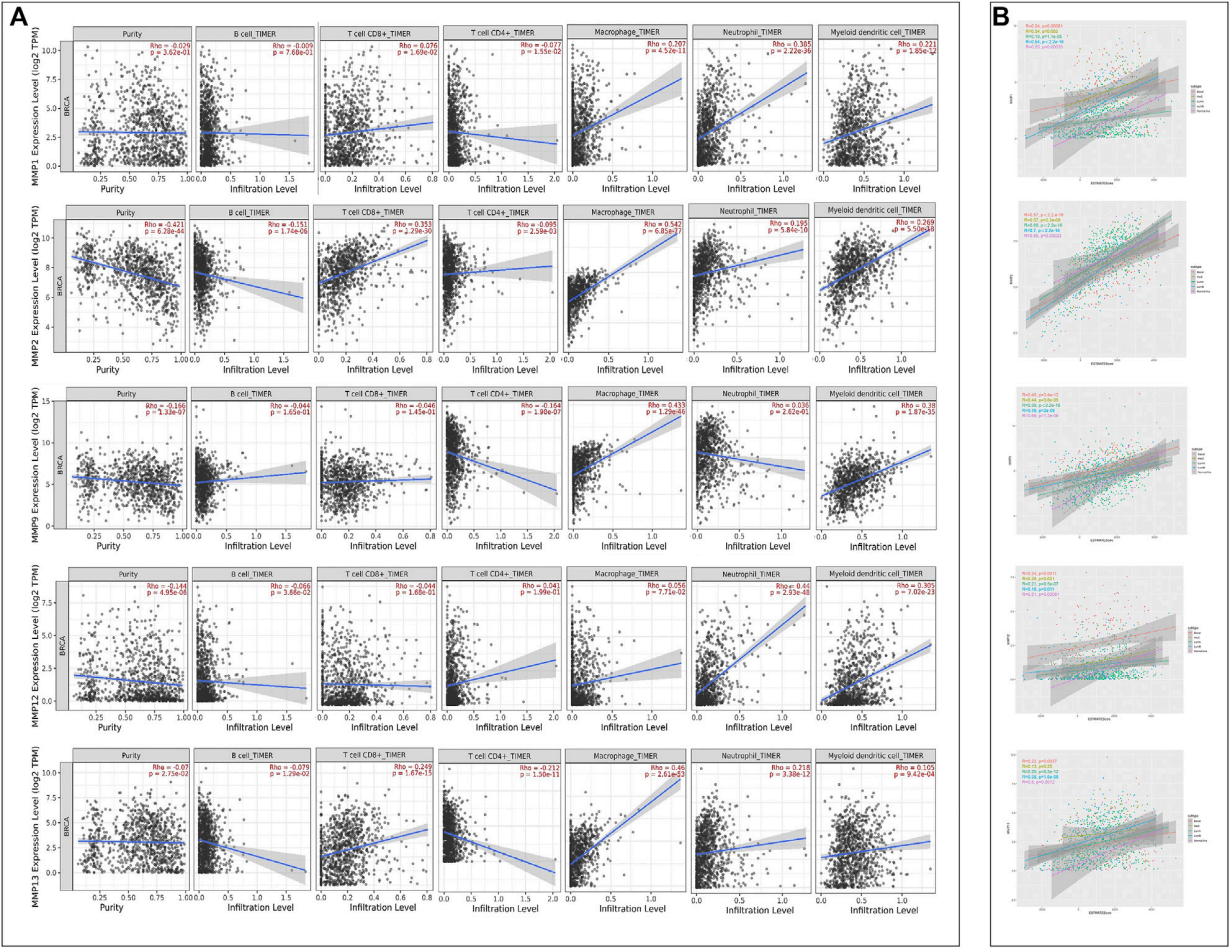
**(A)** Correlations between MMPs expression and immune cell infiltration (TIMER). **(B)** The ESTIMATE Scores of MMPs. The expression levels of MMP1, MMP2, MMP9, MMP12 and MMP13 in five BRCA subtypes were positively associated with the ESTIMATE Scores.

Given the importance of the immune microenvironment on BRCA development, the correlation between MMPs and immune cell infiltration is another concern in the present study. For this purpose, every MMP was imported into Timer2.0 to analyze the correlation between the infiltration of B cells, CD8^+^T cells, CD4^+^T cells, macrophages, neutrophils, and dendritic cells in BRCA, respectively (Table 2 and Figure 8A). MMP1 had a positive correlation with the infiltration of macrophages, neutrophils, and dendritic cells. MMP2, MMP13 and MMP19 were both positively associated with CD8^+^T cells, macrophages, neutrophils, and dendritic cells. Notably, MMP2 and MMP19 expressions were negatively linked to B cells infiltration. In BRCA, MMP13 expression was shown to have a negative relation with CD4^+^T cells infiltration. Regarding MMP9, CD4+T cells, macrophages, neutrophils, and dendritic cells have a positive correlation in BRCA patients. Additionally, the infiltration of neutrophils and dendritic cells was positively associated with MMP12 expression in BRCA. Besides, we have investigated the estimate score of MMP1 and MMP9 in five subtypes of BRCA via Estimate Package. Moreover, we have investigated the stromal score, immune score, and tumor purity of five subtypes, along with the estimate score of MMP1, MMP2, MMP9, MMP12, MMP13 in five subtypes of BRCA. The results showed that estimate scores of five subtypes were positively related with the expression of MMP1, MMP2, MMP9, MMP12, and MMP13 (*p* < 0.05) (Figure 8B).

**TABLE 2:** Correlations between MMPs expression and immune cell infiltration Abbreviations: P, positive; N, negative; 0, without correlation; MMP, Matrix metalloproteinase.

	B Cell	CD8^+^T Cell	CD4^+^T Cell	Macrophage	Neutrophil	Dendritic Cell
MMP1	0	0	0	P	P	P
MMP2	N	P	0	P	P	P
MMP3	N	P	0	P	P	P
MMP7	N	0	P	0	P	P
MMP8	N	0	0	P	P	P
MMP9	0	0	P	P	P	P
MMP10	0	P	N	P	0	0
MMP11	0	P	N	P	0	0
MMP12	0	0	0	0	P	P
MMP13	0	P	N	P	P	P
MMP14	0	P	N	P	P	P
MMP15	0	0	0	0	0	P
MMP16	N	P	N	P	P	0
MMP17	0	0	0	P	N	0
MMP19	N	P	0	P	P	P
MMP20	0	0	P	0	P	P
MMP21	0	P	0	P	0	0
MMP24	0	0	0	N	N	0
MMP25	0	0	P	N	P	P
MMP26	0	0	0	0	N	N
MMP27	N	P	0	P	P	P
MMP28	N	P	0	P	N	P

## 4 Discussion

In recent years, the clinical treatment of BRCA has formed a comprehensive system consisting of surgical treatment, physical therapy, and drug therapy (Harbeck and Gnant 2017). However, in actual clinical application, high mortality, high recurrence rate and adverse reactions are still major problems (Chen et al., 2018). Like other malignant tumors, the metastasis of BRCA is also a multi-step complex process, which includes an important process-cancer cells penetrating the basement membrane (Zhang 2013).

Basement membrane is the extracellular matrix (ECM) constructed by glycoproteins and proteoglycans and is located between the epithelial cell layer and the endothelial cell layer. Benign tumors are restricted to the epithelial cell layer by the basement membrane. Therefore, basement membrane is the first important line of defense against cancer invasion (John and Tuszynski 2001). Once tumor cells undergo malignant transformation, they activate the activity of basement membrane-related proteases through a variety of mechanisms, destroy the structure of the basement membrane, penetrate the basement membrane into the adjacent stroma, and start tumor cell infiltration and metastasis (Condeelis and Segall 2003; Xie and Wang 2005). In this process, MMPs play an important role.

As a family of zinc-dependent proteolytic enzymes, MMPs provide pathways for tumor cell infiltration and metastasis by destroying basement membrane and ECM and damaging the metastatic barrier (Nagase et al., 2006). Therefore, MMPs have become an important factor affecting cancer metastasis, growth and poor prognosis (Egeblad and Werb 2002). In the past studies of MMPs, most of them focused on the expression and prognosis of one or several MMPs in different cancers. There has not been a systematic study of the relationship between whole MMPs and BRCA, along with its five subtypes. Due to the small sample size and some errors of previous studies, the credibility of the conclusions of some independent studies is not high. Therefore, this study is based on bioinformatics and conducts data mining through public biological data platforms to analyze the gene expression and clinical data of BRCA and its subtypes patients, immune cell infiltration and other conditions.

MMP1 belongs to the type of collagenases, which was found to be closely related to lung squamous cell carcinoma (OuYang et al., 2003) and colon cancer (Lu et al., 2011) in previous studies. In this study, GEPIA analysis showed that the expression of MMP1 in BRCA tissues was higher than that in normal tissues. As for subtype analysis, McGowan et al. found that MMP1 was always upregulated (McGowan and Duffy 2008), especially in Her2 and Basal BRCA subtype, which was similar with our results. There are also studies showing that by inhibiting MMP1, bone metastasis in breast cancer patients can be reduced (Lu et al., 2009). Moreover, through the Kaplan-Meier Plotter database, it was found that the high expression of MMP1 can lead to poor prognosis of BRCA. At the same time, among BRCA patients, PIK3CA and TP53 had a high mutation rate (Lu et al., 2019). Through cBioPortal analysis, it was found that MMP1 may be related to the mutations of TP53 and PIK3CA, leading to poor prognosis of breast cancer patients. A Transwell experiment (Wang 2018) proved that when the MMP1 gene was silenced, the invasion and migration ability of BRCA was significantly reduced. In addition, collagen is also an important part of ECM (Ma et al., 2016), while MMP1 can degrade type I, II and III collagen (Singh et al., 2016). This study confirmed that MMP1 participated in collagen metabolism and the degradation of extracellular matrix tissue, promoting cancer metastasis. These results all indicate that MMP1, as an oncogene, might play an important role in the tumorigenesis and progression of BRCA, especially in Her2 and Basel subtype. The high expression of MMP1 in breast cancer tissues made it promising as one of the diagnostic markers of BRCA.

It is also worth noting the role of MMP9 in breast cancer. This study proved that high expression of MMP9 existed in BRCA patients, along with a bad prognosis, and there was a close relationship between the expression level of MMP9 and the pathological stage of the tumor. In an animal study, Mendes et al. (Mendes et al., 2005) found that MMP9 inhibitors significantly reduced the occurrence of breast cancer metastases in brain. As for the subtype analysis, overexpression of MMP9 revealed itself as a prominent feature of Basel and Her2 breast cancers (Yousef et al., 2014), which was almost consistent with our results. Moreover, through TIMER2.0 analysis, it was found that MMP9 had a significant positive correlation with CD4^+^ T cells, dendritic cells, macrophages, and neutrophils, indicating that MMP9 may be involved in the immune cell infiltration process of breast cancer tissues. The microenvironment affects tumor growth. By regulating MMP9, it can interfere with the activities of the tumor microenvironment, thereby inhibiting the metastasis of cancer cells (Si et al., 2021). Even in the urine of BRCA patients, the level of MMP9 was significantly higher than that of normal people (Shen et al., 2010). This is of great significance for breast cancer diagnosis.

In addition to MMP1 and MMP9, it was also found that other MMPs may be related to the prognosis of BRCA in this study. MMP12 had a significant relationship with the pathological stage of breast cancer patients, and patients with high expression levels of MMP12 had a poor prognosis. Studies have shown that CXCR4 mediates MMP12 to degrade the matrix, leading to breast cancer infiltration and growth (Hernandez et al., 2011). MMP15 may increase the level of integrin 6 (ITGB6) by mediating Rho-Rac pathway, leading to breast cancer cell metastasis (Desai et al., 2016), which is consistent with the poor prognosis of breast cancer patients caused by high-level expression of MMP15 in this study. Overexpression of MMP15 had a notably poorer prognosis in Nomal-like subtype patients than that in other subtypes. Besieds, highly expressed MMP7 can reduce the survival rate of breast cancer. In the study of Sizemore et al. (Sizemore et al., 2014), it was found that the high expression of MMP7 and the hypomethylation of the MMP7 promoter can lead to the deterioration of breast tumors. In this study, MMP13 was also highly expressed, which was more prone to connective tissue expression than other MMPs (Vincenti and Brinckerhoff 2002). Among the subtypes, the expression of MMP13 in Her2 and subtype was the highest. Our anlysis also indicated this approximate result (Kim et al., 2014). MMP13 can degrade not only type II collagen and proteoglycan in cartilage, but also type IV and type IX collagen, osteonectin and basement membrane proteoglycan (Shiomi et al., 2010), and participate in the degradation of ECM. At the same time, the overexpression of MMP13 was associated with shortened overall survival of patients (Zhang et al., 2008; Yin and Xia 2013). MMP13 played an important role in the metastasis and invasion of cancer and may be used as one of the prognostic indicators of breast cancer, particularly in Her2 subtype.

In this study of BRCA patients, MMP19 was lowly expressed. The low expression of MMP19 can promote the deterioration and growth of some tumors, such as nasopharyngeal carcinoma. Chan et al. (Chan et al., 2011) believed that MMP19 can inhibit tumors and anti-angiogenesis, but the mechanism of MMP19 for tumor suppression was still unclear. However, the high expression of MMP19 can also lead to the deterioration and poor prognosis of other tumors, such as colorectal cancer (Chen et al., 2019) and glioma (Luo et al., 2018). At present, there are too few studies on MMP19 and breast cancer, and no clear conclusions have been formed. At the same time, this study also found that patients with low MMP19 expression had a poor survival prognosis, and the mechanism needs further study. In addition, TIMER2.0 showed that MMP19 was positively correlated with CD8^+^ T cells, which may be related to pro-inflammatory cytokines (Beck et al., 2008), especially with lymphocyte chemotaxis and interferon-induced T cell a chemotactic agent (I- TAC) related.

The immune cells contained in tumor microenvironment (TME) had cancer-promoting and anti-tumor effects, which can affect the progression and recurrence of tumors (Zhou et al., 2021). Analysis of the correlation between MMPs and six types of immune cell infiltration through TIMER2.0 proved that MMPs were not only related to the prognosis of breast cancer, but also reflected the immune cell infiltration status of tumor cells. Regarding the analysis of subtypes, our results showed that estimate scores of five subtypes were positively related with the expression of MMP1, MMP2, MMP9, MMP12, and MMP13. Our results showed that low tumor purity of BRCA subtypes had a higher malignancy and worse prognosis, whereas had stronger immunophenotypes, which was consistent with some studies of glioma tumors (Zhang et al., 2017) and colon cancer (Mao et al., 2018).

In addition, we discovered through the STRING database and the GeneMANIA database that MMPs were related to the synthesis and secretion pathway of parathyroid hormone, IL-17 signaling pathway, relaxin signaling pathway, the process of collagen metabolism, and the effect on the activity of serine hydrolase, serine peptidase, and aminopeptidase: 1) Breast cancer patients produce more parathyroid hormone than normal people (Li et al., 2010), leading to hypercalcemia, and promote breast cancer invasion and bone metastasis through autocrine, paracrine and exocytosis mechanisms (Tovar Sepulveda and Falzon 2002). 2) As a cytokine, interleukin 17 (IL-17) relates to several inflammatory diseases with its pleiotropic effects (Bettelli et al., 2007). Obradović et al. investigated that the increase of MMP9 was stimulated by IL-17 via downregulating ERK1/2 activation (Obradović et al., 2016). It was also indicated that IL-17 could induce elevated expression of MMP-1 via p38 MAPK- and NF-kappaB, ERK-dependent AP-1 activation (Cortez et al., 2007). As previously described, IL-17 had a prominent effect on the modulation of MMP (Jovanovic et al., 2000; Singh et al., 2018). 3) In previous studies, relaxin has a close connection with MMPs improving the tumor metastasis. The study of Chow et al. investigated the extent that the NO pathway was involved in positive functions of relaxin regulating MMP1, MMP2, MMP9 and MMP13 (Chow et al., 2012). Ma et al. provided the evidence that relaxin siRNA could down-regulate the expression of MMP9, thus promoted the proliferation, invasion, and metastasis of tumor cells (Ma et al., 2013). The mechanism of enhanced invasiveness may lie in the formation of MT1-MMP-enriched invadopodia by overexpression of relaxin (Bialek et al., 2011). 4) Collagen IV is the main component of the extracellular matrix, so its degradation is related to the infiltration and spread of malignant tumors. Related studies have shown that the high expression of MMP2 and MMP9 is positively correlated with the degradation of collagen IV (Ma et al., 2016). 5) According to Jun research (Jun et al., 2020), ABHD12 is a serine hydrolase that can inhibit the growth, proliferation, migration, and invasion of breast cancer cells. 6) Abnormal overexpression of human kallikrein-related peptidase 6 (KLK6) among serine peptidases is one of the characteristics of a subset of breast cancer. Pampalakis’ experiments (Pampalakis et al., 2019) showed that its overexpression was related to the increased ability of breast cancer cells to metastasize to the lung, the increased expression of certain S100 proteins and keratins, and the down-regulation of apoptosis-related proteases 6. Feng et al. (2016) (Feng et al., 2016) used the immunohistochemical SP method to detect the expression of aminopeptidase in 80 cases, and found that the positive expression rate of aminopeptidase in breast cancer was much higher than that of normal breast tissue adjacent to cancer. These six points are consistent with the research of this article. MMPs may affect the above six points and cause the invasion and metastasis of breast cancer.

## 5 Conclusion

MMPs play multiple biological roles in the pathogenesis and development of breast cancer. MMP1 and MMP9 can be used as independent prognostic factors to predict the prognosis of breast cancer patients, in order to further study the pathological mechanism and possible treatment targets for breast cancer, especially for Her2 and Basel subtypes.

## Data Availability

The original contributions presented in the study are included in the article/Supplementary Material, further inquiries can be directed to the corresponding author.

## Abbreviations

BRCA, Breast cancer; ECM, Extracellular matrix; Her2, Human epidermal growth factor receptor 2-positive/estrogen receptor-negative (HER2+/ER−); MMPs, Matrix metalloproteinases; OS, Overall survival; PPI, Protein-protein interactions; RFS, Recurrence-free survival; TME, Tumor microenvironment.
